# The effect of ultrasound and freezing/thawing treatment on the physical properties of blueberries

**DOI:** 10.1007/s10068-018-0528-5

**Published:** 2018-12-08

**Authors:** Konrad W. Nowak, Magdalena Zielinska, Katarzyna M. Waszkielis

**Affiliations:** Department of Systems Engineering, Faculty of Technical Sciences, University of Warmia and Mazury in Olsztyn, Heweliusza 14, 10-719 Olsztyn, Poland

**Keywords:** Ultrasound treatment, Freezing, Texture properties, Color, Blueberries

## Abstract

The aim of the study was to investigate the effect of ultrasound treatment and freezing/thawing on the physical properties of blueberries (*Vaccinium corymbosum L.*). Fruits were subjected to ultrasound treatment, mechanical freezing/thawing, and freezing/thawing with subsequent ultrasound treatment. Moisture, density, porosity, hardness, springiness, cohesiveness, chewiness, gumminess, and color of blueberries were analyzed. Ultrasound treatment and freezing/thawing significantly decreased particle density and increased porosity of blueberries (*p *< 0.05). Ultrasound treatment and freezing/thawing produced significantly softer, less chewy and gummy berries in relation to control sample (*p *< 0.05). All techniques induced considerable changes in the color of blueberries. The results indicates that ultrasound treatment performed after freezing/thawing, didn’t exert any effect on the fruits in relation to freezing/thawing alone (*p *> 0.05); however, it is an interesting technique for processing fresh blueberries and an alternative to freezing/thawing, when the preservation of product quality is a priority or when rapid textural damage is required before diffusion processes.

## Introduction

Blueberries (*Vaccinium corymbosum* L.) are widely consumed perishable fruits which are rich in anthocyanins, bioactive compounds that are responsible for the desirable reddish blue color of blueberry fruits; therefore, color measurement can provide useful information about the content of these compounds (Rolle and Guidoni, 2007; Zielinska and Michalska, 2016). Due to their seasonal and perishable nature, blueberries have to be preserved in some form for later consumption. Fruits with wax-coated skins, such as blueberries, require special treatment to increase the permeability of the surface layer to heat and moisture transfer. Traditional methods of initial pretreatment include freezing/thawing of blueberries, which significantly reduces drying time and specific energy consumption of drying relative to drying without initial pre-treatment (Feng et al., 1999; Zielinska et al., 2015). This method also significantly decreases cuticle thickness (to approximately half of the initial value). Freezing/thawing significantly lowers the maximum force and the work required to puncture the skin of the processed berries relative to raw fruits. However, the combination of freezing/thawing and drying adversely affects the quality of the final product (Zielinska et al., 2015). Even the freezing itself can also decrease an amount of anthocyanins (Vollmannova et al., 2009). In addition to traditional food processing methods, a number of new techniques have been proposed to, improve the physicochemical properties of food products, and enhance processing efficiency (e.g., by increasing the drying rate, minimizing energy consumption and shortening processing time). In the group of the emerging technologies, such as ultra-high pressure processing (HPP), microwave (MW), high intensity pulsed electric field (PEF) and pulsed X-ray processing, ultrasound treatment shows particular promise for processing specific food materials, including fruits and vegetables (Fijalkowska et al., 2015; Pakbin et al., 2015). High-energy (high-power, high-intensity) ultrasound treatment with a frequency of 18–100 kHz and intensity higher than 1 W/cm^2^ (typically between 10 and 1000 W/cm^2^) can be used as an initial pretreatment method or the main method of different food products preservation (McClements, 1995). High-power ultrasound treatment can induce chemical and physical changes in food products due to acoustic cavitation. A local increase in temperature in the vicinity of collapsing bubble “hot spots”, changes in pressure induced by shockwave emissions or even surface pitting promote various chemical and physical changes, and lead to cell damage (Chandrapala et al., 2012). The compression and expansion of a material (referred to as the sponge effect) lead to the formation of micro channels in cells and causes leakage of intracellular liquid to the surrounding area (Farhanineyad et al., 2017). Compared to traditional methods, increased heat and mass transfer processes can be observed during ultrasound treatment (Ensminger, 1988; Li et al., 2010). However, it depends on the treatment time, ultrasound intensity and type of material. The retention of chemical compounds (anthocyanins, ascorbic acid) after ultrasound treatment is also higher relative to thermal processing (Tiwari et al, 2008; Tiwari et al, 2009). However, ultrasound treatment may exert both desirable and undesirable effects on the nutritional and physicochemical properties of food products. Therefore, the influence of ultrasound treatment on the nutritional value and the technological, functional and sensory attributes of foods should be evaluated individually for each product (Soria and Villamiel, 2010). Ultrasound has been applied as a preliminary treatment before or during processing (like dehydration or extraction) to modify properties and susceptibility to heat and mass transfer of various fruits and vegetables such as apples, bananas, melons, pineapples, papayas, mushrooms, Brussel sprouts, cauliflower sprouts, grapes (and grape wastes), olives (and olive wastes), wild garlic, pomegranates, grapefruits, potatoes, black carrot, cranberries and many others (Aydar, 2018; Li et al., 2010; Medina-Torres et al., 2017; Mothibe et al., 2011; Wiktor et al., 2015; Zielinska and Markowski, 2017). Despite the above, the effect of ultrasound treatment on the physical properties of blueberry fruits has not yet been described.

The aim of the present study was to investigate the effect of ultrasound pre-treatment (applied to raw and frozen/thawed fruits) on the particle density, porosity, hardness, springiness, cohesiveness, chewiness, gumminess and color of blueberries (*Vaccinium corymbosum L.*), and to compare the results with raw and frozen/thawed blueberry samples.

## Materials and methods

### Material

Polish-grown blueberry fruits (*Vaccinium corymbosum L.*) were purchased in a local market. Fruits were fresh, ripe and firm. They were divided into four groups and packed in sealed plastic containers immediately after purchase. The first group was subjected to ultrasound treatment, the second group was subjected to freezing/thawing treatment, the third group was frozen/thawed and ultrasound-treated, and the fourth group was the control (untreated) sample. Ultrasound treatment and freezing and thawing of samples is described below.

### Moisture content analysis

The moisture content of berries was evaluated by the air-oven drying method (using Binder FED53 127 heating chamber, BINDER GmbH, Tuttlingen, Germany) according to the applicable standard (AOAC, 1975). Oven temperature was set to 105 °C and heating time was 24 h. The results were expressed as the mean value of three replications.

### Ultrasound treatment

Blueberry fruits were subjected to ultrasound treatment (ultrasound frequency: 45 kHz; time: 2 min) in the EMMI 55HC-Q ultrasound bath (EMAG AG, Mörfelden-Walldorf, Germany) equipped with six ultrasound transducers. Bath volume was 9 dm^3^ and total ultrasonic power was 300 W, which is equivalent to 33.3 W/dm^3^. Blueberry samples of 0.2 kg each were placed in perforated polyethylene terephthalate (PET) containers and immersed in the center of the water bath. The water had room temperature (20 ± 1 °C) which increased by around 2.7 °C during 2 min of processing.

### Freezing/thawing treatment

Blueberries were frozen mechanically at a temperature of − 24 ± 1 °C with the estimated freezing rate of 2.55 °C min^−1^, and were freeze-stored for 24 h in the Liebherr GT 4932 chest freezer (Liebherr-Hausgeräte GmbH, Ochsenhausen, Germany) with 449 dm^3^ capacity. Frozen blueberries were thawed on an open tray for around 1 h to reach equilibrium temperature (20 ± 2 °C) before mechanical tests.

### Determination of true density, particle density and porosity

The true density of the dry substance, ρ_ds_, was initially determined by the liquid pycnometer method (Odeniyi et al., 2011). Blueberries were dehydrated at 105 °C for 24 h in an air-oven (Binder FED53 127, BINDER GmbH, Tuttlingen, Germany) according to the applicable standard (AOAC, 1975) and were ground in a laboratory mill. Approximately 2 g of the dried sample was used per experiment. True density was determined with a non-water-miscible liquid (xylene) and a calibrated glass pycnometer of approximately 50 ml. Xylene density at 20 °C was determined at 864 ± 1 kg/m^3^. The true density of the dry substance of blueberries was calculated using the following formula:

1 $$\rho_{Tds} = \frac{{864 \cdot \left( {m_{3} - m_{1} } \right)}}{{m_{2} + \left( {m_{3} - m_{1} } \right) - m_{4} }}$$

where *ρ*_*Tds*_ is the true density of the dry substance (kg/m^3^), *m*_*1*_ is the mass of an empty pycnometer (kg), *m*_*2*_ is the mass of the pycnometer with the reference fluid (non-solvent) (kg), *m*_*3*_ is the total mass of the pycnometer and the sample (kg), and *m*_*4*_ is the total mass of the pycnometer with the non-solvent and the sample (kg).

The true density of blueberry fruits with a given moisture content was calculated with the following formula (Sahin and Sumnu, 2006):

2 $$\rho_{T} = \frac{1}{{\mathop \sum \nolimits_{i = 1}^{n} \frac{{x_{i} }}{{\rho_{i} }}}}$$

where *ρ*_*T*_ is the true density of blueberry fruits with a given moisture content (kg/m^3^), *x*_*i*_ is the weight fraction of the *i*th component (water and dry matter), and *ρ*_*i*_ is the density of the *i*th component (kg/m^3^) which, in this case, was the density of water at the analyzed temperature and the true density of the dry substance (*ρ*_*Tds*_) determined above.

Particle density, *ρ*_*p*_, was calculated from the mass and volume of the sample measured by the solid displacement method (Rahman, 1995). Quinoa seeds were used as the solid material. The density of quinoa seeds was determined at 747 ± 6 kg/m^3^ from the measured volume and mass of the seeds (Zielinska et al., 2015). True density and particle density values were averaged over 6 measurements.

Porosity, *ε*, defined as the ratio of total enclosed air space or void volume to the total volume of a material, was calculated using the following equation (Rahman, 1995):

3 $$\varepsilon = \left( {1 - \frac{{\rho_{p} }}{{\rho_{T} }}} \right)$$

where *ε* is porosity, –, *ρ*_*p*_ is particle density, kg/m^3^, and *ρ*_*T*_ is true density, kg/m^3^.

### Determination of textural properties

In the texture profile analysis (TPA), blueberry fruits were compressed twice in the TA.HD Plus Texture Analyzer (Stable Micro Systems, Godalming, UK), and their hardness, springiness, cohesiveness, chewiness and gumminess were determined from the force deformation curve using method similar to Chong et al. (2013). During the test a flat 50 mm-diameter piston was used, the test speed was 2 mm/s, and strain was 50% of sample height. The results were averaged over thirty measurements.

### Microscopic observations

Blueberry fragments were dried by critical point drying (CPD 030, BAL-TEC, Balzers, Liechtenstein). The following procedure was adopted to prepare the samples for drying: (1) the fruits were cut perpendicular to the long axis with a fresh razor blade, (2) each sample was placed in a fixative containing glutardialdehyde (2.5 g = 100 g in 0.1 mol/L phosphate buffer, pH = 7.2) for 48 h at a temperature of 4 °C, (3) the sample was rinsed in Milli-Q water, and (4) the sample was dehydrated for 15 min in a graded ethanol series (30–99.8 mL = 100 mL). The specimens were mounted on aluminum stubs using silver paste and coated with gold in a vacuum evaporator (JEE 400, JEOL, Tokyo, Japan) to make their surfaces electrically conductive for SEM analysis. The micrographs of berry cross-sections were acquired with a scanning electron microscope (JEOL, model 5200, Tokyo, Japan), and accelerating voltage was set at 10 kV.

### Color measurement

The color of raw, ultrasound-treated, frozen/thawed as well as frozen/thawed and ultrasound-treated blueberries was determined using the MiniScan XE Plus spectrophotometer (Hunter Associates Laboratory Inc., Reston, VA, USA) with standard illuminant D 65, 10° observer and 8° diaphragm. Blueberry samples with a weight 0.020 ± 0.001 kg each, were placed in a container with a fixed shape and size, and color was determined directly on the surface of the fruits. The results were averaged over 35 measurements for each sample.

The display was set to CIELAB color coordinates, where L* indicated lightness or darkness, a* indicated redness (+) or greenness (−), and b* indicated yellowness (+) or blueness (−). Based on the changes in the individual color parameters of blueberries, the total change in color (∆E*) during freezing/thawing and ultrasound treatment was calculated from Eq. 4 (Hutchings, 1999):

4 $$\Delta E^{*} = \sqrt {(\Delta L^{*} )^{2} + (\Delta a^{*} )^{2} + (b^{*} )^{2} }$$

where:

5 $$\Delta L^{*} = L^{*}_{\text{Control}} - L^{*}_{\text{Sample}}$$

6 $$\Delta a^{*} = a^{*}_{\text{Control}} - a^{*}_{\text{Sample}}$$

7 $$\Delta b^{*} = b^{*}_{\text{Control}} - b^{*}_{\text{Sample}}$$

The total difference in color was classified as highly distinct (∆E* >* 3*), distinct (1.5 < ∆E* <* 3*), and unnoticeable (∆E* <* 1.5*) (Tiwari et al., 2008).

### Statistical analysis

The differences between samples were determined by the Mann–Whitney U-test at a confidence level of 95% (*p *< 0.05). The calculations were performed in Statistica 9.0 software (StatSoft Inc., Tulsa, OK, USA).

## Results and discussion

### Results of the moisture content analysis

The initial moisture content of raw blueberries was 8.75 kg H_2_0/kg db (dry basis), what corresponds to 89.7% of moisture. The significant differences in the moisture content, density and porosity of raw and ultrasound-treated blueberries (*p *< 0.05) (Table 1) can be attributed to the physicochemical changes induced by freezing/thawing and the resulting loss of moisture. Total moisture loss during freezing/thawing was estimated at 3.3 ± 0.6%, and it resulted from the loss of water vapor under exposure to the cooling medium. The moisture content of frozen/thawed blueberries was 8.46 kg H_2_0/kg db, what corresponds to 89.4% of moisture. Qualitative changes caused by the formation, growth and thawing of ice crystals contributed to total moisture loss as well as significant changes in the physical properties of blueberries (*p *< 0.05), which is discussed below. Ultrasound treatment did not exert a significant influence on the moisture content of blueberry fruits or the moisture content after freezing/thawing in comparison to frozen/thawed blueberries (*p *> 0.05). The moisture content of ultrasound-treated blueberries was 8.70 kg H_2_0/kg db and 8.44 kg H_2_0/kg db for frozen/thawed and ultrasound-treated fruits, respectively, what corresponds to 89.7% and 89.4 of moisture respectively.

**Table 1:** Moisture content, particle density and porosity of raw blueberries and blueberries subjected to three different treatments (the mean and standard error of the mean)

Sample	Moisture content, MC (kg H_2_0/kg db)	Particle density, ρ_p_ (kg/m^3^)	Porosity, ε (−)
Raw	8.75 ± 0.03^a^	992 ± 18^a^	0.062 ± 0.017^a^
Ultrasound-treated	8.70 ± 0.06^a^	919 ± 21^b^	0.135 ± 0.020^b^
Frozen/thawed	8.46 ± 0.04^b^	860 ± 21^c^	0.191 ± 0.020^c^
Frozen/thawed and ultrasound-treated	8.44 ± 0.05^b^	857 ± 20^c^	0.194 ± 0.022^c^

Identical letters in the same column indicate that the mean values do not differ significantly at a confidence level of 95% (*p *< 0.05)

### Results of density and porosity analysis

Martynenko (2014) developed a method for evaluating the true density, particle density and porosity of shrinkable biomaterials based on the results of drying experiments and determined the true density, initial particle density and initial porosity of blueberries with initial moisture content of 9.8 kg H_2_0/kg db at 1610 kg/m^3^, 1035 kg/m^3^ and 0.044, respectively. In the present study, the particle density of raw blueberries was determined at 992 kg/m^3^. True density was estimated at 1553 kg/m^3^ based on the chemical composition of blueberries. The porosity of raw blueberries was determined at 0.062 based on the obtained values of true density and particle density. The effect of ultrasound treatment and freezing/thawing on the particle density and porosity of blueberries was evaluated, and the results are shown in Table 1. The particle density of ultrasound-treated blueberries was 919 kg/m^3^, and it was significantly lower (*p *< 0.05) than that of raw fruits. Freezing/thawing treatment and freezing/thawing with ultrasound treatment induced more significant changes in particle density than the ultrasound treatment of fresh fruits. Particle density was determined at 860 kg/m^3^ for frozen/thawed blueberries and at 857 kg/m^3^ for fruits subjected to freezing/thawing with ultrasound treatment, and it was significantly lower (*p *< 0.05) than that of raw and ultrasound-treated fruits. The observed changes in particle density were accompanied by statistically significant (*p *< 0.05) differences in sample porosity after ultrasound treatment, freezing/thawing and after freezing/thawing with ultrasound treatment. The porosity of ultrasound treated blueberries was determined at 0.135, and it was significantly higher (*p *< 0.05) than that of raw fruits. Similarly to particle density, more significant changes in sample porosity were observed after freezing/thawing treatment, both with or without ultrasound treatment, than after ultrasound treatment of fresh fruits. As a result, the highest porosity values were observed in frozen/thawed fruits (0.194) and in fruits subjected to freezing/thawing with ultrasound treatment (0.191) (Table 1).

Despite the similar moisture content of raw and ultrasound-treated samples (non-significant differences, *p *> 0.05), the samples subjected to ultrasound treatment were characterized by significantly lower particle density and higher porosity (*p *< 0.05) than raw fruits. The above could be attributed to the micro-puffing of fruit cells caused by the expansion and evaporation of water, due to the energy released by collapsing bubbles during acoustic cavitation, that leads to the formation of hot-spots, which can increase temperature up to 5000 K and pressure up to 500 atm (Suslick, 1990). As a result, ultrasound treatment probably caused irreversible damage to cell membranes. Most probably, the main factors responsible for cell damage were the mechanical forces resulting from the collision of cavitation bubbles, shock waves caused by bubble implosion or microstreaming resulting from changes in bubble size. Despite the above, the absence of significant moisture loss during ultrasound treatment (*p *> 0.05) indicates that the surface layer was still impermeable to water, probably because acoustic cavitation was not strong enough to cause skin lesions; therefore, ultrasound processing did not produce fissures and microcracks in blueberry fruits.

The qualitative changes induced by the formation and recrystallization of ice crystals during the freezing and subsequent thawing of blueberries significantly decreased the density and increased the porosity of the processed samples relative to raw fruits. Ice crystals formed during slow mechanical freezing probably induced significant changes in the structure of blueberries, which led to severe cell damage and water loss (Delgado and Rubiolo, 2005). According to the literature, moisture loss during freezing and thawing is caused mainly by cell wall deterioration, which leads to decrease in the thickness of the surface layer (Zielinska et al., 2015) and the loss of the ability to act as a semipermeable membrane or diffusion barrier (Delgado and Rubiolo, 2005). Frozen/thawed fruits have a thinner surface layer than control sample, and they are not protected against moisture loss during thawing.

### Results of mechanical properties measurement

**Fig. 1:**
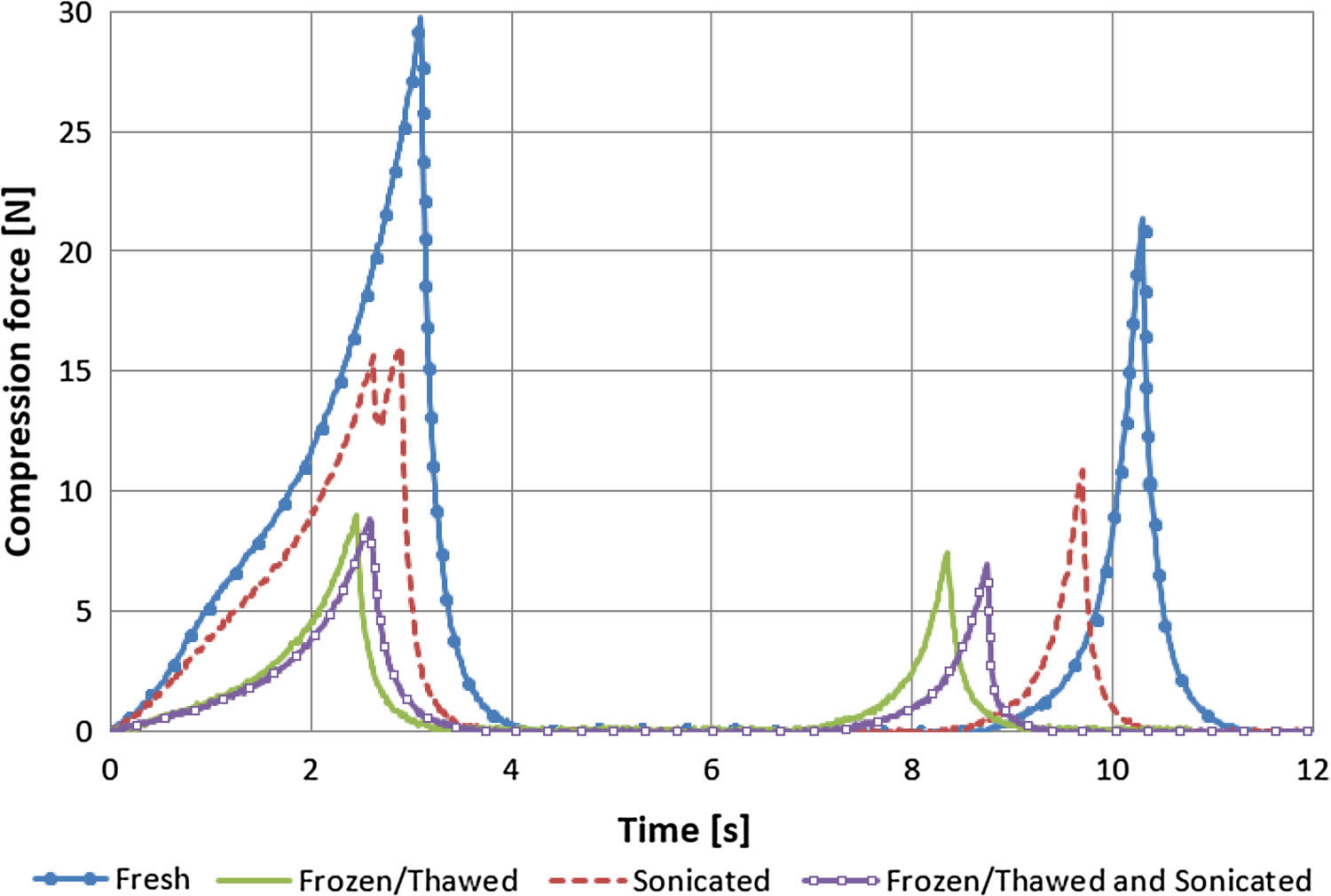
A force deformation curve for raw (untreated), ultrasound-treated (sonicated), frozen/thawed, and frozen/thawed and ultrasound-treated (sonicated) blueberries

The effects of freezing/thawing, ultrasound treatment and freezing/thawing followed by ultrasound treatment on the mechanical properties of blueberries are presented in Table 2. Additionally, selected results of the TPA test performed on raw, ultrasound-treated and frozen/thawed fruits as well as blueberries subjected to freezing/thawing followed by ultrasound treatment are shown in Fig. 1. Non-treated berries were much more rigid and more difficult to deform than processed fruits. Control sample required the highest maximum compression force which was determined at 29.1 N.

**Table 2:** The results of the Texture Profile Analysis of raw blueberries and blueberries subjected to three different treatments (the mean and standard error of the mean

Sample	Hardness (N)	Springiness (−)	Cohesiveness (−)	Gumminess (N)	Chewiness (N)
Raw	29.1 ± 0.6^a^	0.44 ± 0.01^a^	0.168 ± 0.007^a^	4.91 ± 0.23^a^	2.21 ± 0.14^a^
Ultrasound-treated	16.0 ± 0.5^b^	0.39 ± 0.01^b^	0.147 ± 0.004^b^	2.38 ± 0.12^b^	0.94 ± 0.06^b^
Frozen/thawed	9.1 ± 0.4^c^	0.43 ± 0.02^a,b^	0.190 ± 0.024^a,b^	1.83 ± 0.28^c^	0.93 ± 0.18^b^
Frozen/thawed and ultrasound-treated	9.4 ± 0.5^c^	0.43 ± 0.02^a,b^	0.190 ± 0.021^a,b^	1.80 ± 0.24^c^	0.89 ± 0.16^b^

Identical letters in the same column indicate that the mean values do not differ significantly at a confidence level of 95% (*p *< 0.05)

The hardness of frozen/thawed berries was determined at 9.1 N, and it was significantly lower (more than three-fold, *p *< 0.05) in comparison with control sample. In consequence, the chewiness and gumminess of frozen/thawed berries were more than twice lower than that of raw fruits. Presumably, ice crystals formed during freezing disrupted fruit cell walls and induced significant changes in fruit texture. The springiness and cohesiveness of blueberries remained fairly constant during freezing/thawing at 0.43 and 0.190, respectively. The chewiness and gumminess of frozen/thawed fruits were determined at 0.93 and 1.83, respectively (Table 2).

**Fig. 2:**
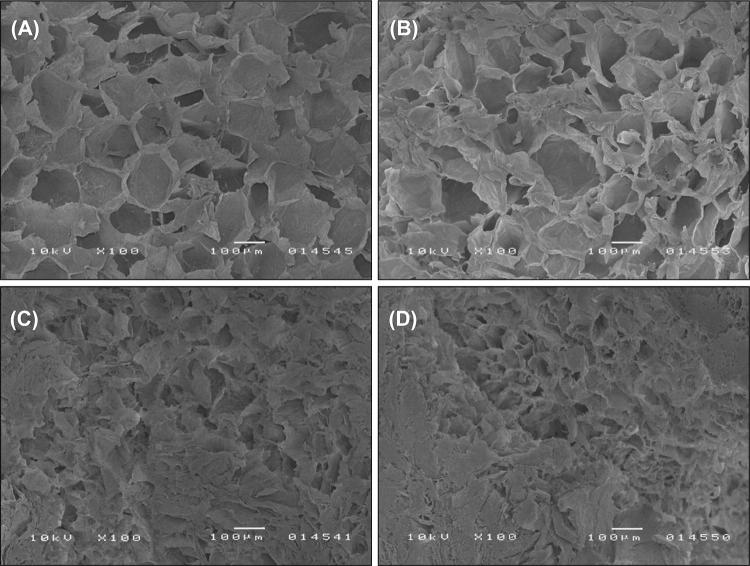
The microstructure of: (**A**) raw (untreated), (**B**) ultrasound-treated, (**C**) frozen/thawed, (**D**) frozen/thawed and ultrasound-treated blueberries

Ultrasound treatment also significantly decreased the hardness of blueberries (*p *< 0.05), but the observed decrease was not as profound as that induced by freezing/thawing and by freezing/thawing followed by ultrasound treatment. The hardness of ultrasound-processed berries was determined at 16.0 N, and it was nearly half that of raw fruits. In comparison with raw fruits, ultrasound-treated blueberries were characterized by significantly lower springiness (11%), chewiness (52%) and gumminess (57%) (*p *< 0.05). The cohesiveness of blueberries remained fairly constant during ultrasound treatment. The springiness, cohesiveness, chewiness and gumminess of ultrasound-processed fruits were determined at 0.39, 0.147, 0.94 N and 2.38 N, respectively (Table 2). The textural changes induced by ultrasound treatment certainly were caused by acoustic cavitation which disrupted cell membranes and contributed to their flaccidity (Fig. 2A); however, the observed changes were less pronounced than in frozen/thawed fruits (Fig. 2C and D). Ultrasound treatment exerted a greater influence on fruit springiness and cohesiveness (changes in cohesiveness were not significant, *p *> 0.05) than freezing/thawing and freezing/thawing followed by ultrasound treatment. Acoustic cavitation probably led to the formation of hot-spots with a very high temperature (Suslick, 1990) which induced microscopic changes in fruit tissue and caused irreversible changes in fibers, the main building blocks of cell walls. High temperature can degrade both pectin (above 180 °C) and cellulose (above 230 °C), decrease fiber strength and lead to the formation of defects inside the cell wall (Baley et al., 2012), which could explain why the initial structure of fruit tissue was not restored after the first compression cycle.

The hardness of frozen/thawed blueberries subjected to ultrasound treatment was determined at 9.4 N, and ultrasound treatment exerted a less significant effect (*p *> 0.05) on fruit hardness than freezing/thawing alone (Table 2). In comparison with frozen/thawed fruits, ultrasound treatment had no significant influence (*p *> 0.05) on fruit springiness (0.43), cohesiveness (0.190), gumminess (1.80 N) and chewiness (0.89 N), either. The above could be attributed mainly to the massive structural deformations caused by freezing/thawing treatment (Fig. 2C), whereas noticeable changes were not observed in ultrasound-processed blueberries (Fig. 2D). The microstructure of fruits subjected to freezing/thawing with ultrasound treatment is nearly identical to that of blueberries that were frozen/thawed only (Fig. 2). Similar results were reported by Lyng et al. (1998) who studied other biological materials with high water content (heifer and lamb meat) and demonstrated (unintentionally, because all samples, ultrasonically treated and non-treated were frozen before mechanical testing) that ultrasound treatment and freezing/thawing did not exert a significant influence on texture parameters, such as bite force, in comparison with freezing/thawing alone. To summarize, ultrasound treatment, freezing/thawing and freezing/thawing followed by ultrasound treatment significantly decreased particle density and increased the porosity of blueberries (*p *< 0.05). Processed fruits were softer, less chewy and less gummy than control sample, which was most evident in frozen/thawed berries and in fruits that were ultrasound processed after freezing/thawing.

### Results of color measurement

The effects of ultrasound treatment, freezing/thawing and freezing/thawing followed by ultrasound treatment on the color attributes of blueberries are presented in Table 3. The lightness (L*), redness (a*), and blueness (b*) of raw blueberries were determined at 33.2, − 0.15 and − 0.45, respectively. These values are in the range found in literature (Matiacevich et al., 2013), where typically values of a* are between − 5 and 5, and for b* from − 10 to 5. Ultrasound treatment induced significant differences only in the value of parameter L* (*p *< 0.05), whereas the a* and b* values of ultrasound-processed fruits did not change significantly, which indicates that ultrasound treatment preserves the desirable reddish–blue color of blueberries brought on by anthocyanins. Our results suggest that anthocyanin degradation was relatively low probably due to the short time of ultrasound treatment. It seems that ultrasound treatment may be considered as a potential technique of initial pretreatment when the preservation of a product’s nutritional quality is a priority, what is similar to observations made by Tiwari et al. (2009). Frozen/thawed blueberries were darker, more red and less blue than raw fruits. The values of parameters L*, a* and b* changed significantly during freezing/thawing (*p *< 0.05), which contributed to changes in red and blue regions. The lightness (L*), redness (a*), and blueness (b*) of ultrasound-processed fruits were determined at 31.5, − 0.10 and − 0.34, respectively, whereas the lightness (L*), redness (a*), and blueness (b*) of frozen/thawed blueberries were determined at 31.0, 0.53 and 0.53, respectively. Similarly to freezing/thawing, ultrasound treatment after freezing/thawing produced darker fruits that were even more red and less blue than the blueberries subjected to freezing/thawing alone. The lightness (L*), redness (a*) and blueness (b*) of frozen/thawed and ultrasound-treated blueberries were determined at 30.6, 1.15 and 0.03, respectively. However, it should be noted that the changes in color identified during instrumental evaluations may not be visible to the naked eye (Gomez-Lopez et al., 2010). The total changes in color (∆E*), calculated based on the changes in individual color parameters of blueberries subjected to ultrasound treatment, freezing/thawing and freezing/thawing followed by ultrasound treatment, were determined at 1.70, 2.29, and 2.88, respectively. Based on the results reported by Tiwari et al. (2008), the total changes in color induced by ultrasound treatment and freezing/thawing (with or without subsequent ultrasound treatment) were considered as distinct (1.5 < ∆E* <* 3*). To summarize, ultrasound-treated fruits were significantly darker than raw (untreated) berries (*p *< 0.05), but significant differences in their a* and b* values (*p *> 0.05) were not observed, which indicates that ultrasound treatment preserves the desirable reddish–blue color brought on by anthocyanins. Frozen/thawed blueberries were darker, more red and less blue than raw (untreated) fruits. The values of parameters L*, a* and b* changed significantly during freezing/thawing, which contributed to changes in red and blue regions. The ultrasound treatment performed after freezing/thawing merely worsen undesirable changes in color and did not influence the rest of properties of frozen/thawed fruits.

**Table 3:** The color of raw blueberries and blueberries subjected to three different treatments (the mean and standard error of the mean are given in brackets)

Sample	L*	a*	b*	∆E*
Raw	33.2 ± 0.3^a^	− 0.15 ± 0.06^a^	− 0.45 ± 0.14^a^	0.00 ± 0.00^a^
Ultrasound-treated	31.5 ± 0.2^b^	− 0.10 ± 0.06^a^	− 0.34 ± 0.06^a^	1.70 ± 0.61^b^
Frozen/thawed	31.0 ± 0.3^b,c^	0.53 ± 0.07^b^	0.53 ± 0.07^b^	2.29 ± 0.80^b^
Frozen/thawed and ultrasound-treated	30.6 ± 0.3^c^	1.15 ± 0.15^c^	0.03 ± 0.22^c^	2.88 ± 0.77^b^

Identical letters in the same column indicate that the mean values do not differ significantly at a confidence level of 95% (*p *< 0.05)

This study demonstrated that using high power ultrasound with power level 33.3 W dm^−3^ or lower on previously frozen and thawed fruits is unjustified, because then ultrasound treatment induce only undesirable changes in the color of blueberries, without any additional effect on the texture.

The results of this study indicate that ultrasound treatment is an interesting technique for processing fresh blueberries, that offers an alternative to time- and energy-consuming freezing/thawing treatments. Ultrasound treatment is particularly dedicated when the preservation of high product quality is a priority and when rapid processing methods are required.
